# Impact of systematic urinary catheterization protocol in delivery room on covert postpartum urinary retention: a before-after study

**DOI:** 10.1038/s41598-017-18065-8

**Published:** 2017-12-18

**Authors:** Mathias Neron, Lucie Allègre, Stéphanie Huberlant, Eve Mousty, Renaud de Tayrac, Brigitte Fatton, Vincent Letouzey

**Affiliations:** Department of Obstetrics and Gynecology, Nîmes University Hospital, Nîmes, France

## Abstract

We investigated whether implementation of a routine catheterization procedure in labor improves covert postpartum urinary retention (cPUR) rates. We conducted a prospective before-after study. 121 women admitted to delivery room in the observational group, and 82 in the intervention group, in a tertiary university hospital in Southern France were included. All patients in the intervention group were systematically catheterized 2 hours after delivery. cPUR was screened for in both groups. The primary end-point was cPUR (post-void residual bladder volume >150 ml when voided volume is >150 mL). The rate of cPUR decreased from 50% (60 out of 121 patients) in the observational group to 17% (14/82) in the intervention group (OR = 0.21; 95% Confidence Interval [0.13;0.58]; p < 0.001). Similarly, in the subgroup of patients who underwent instrumental delivery, the rate of cPUR was lower in the intervention group (18%, 2/11) than in the observational group (65%, 15/23) (p = 0.02). Systematic intermittent bladder catheterization immediately postpartum could decrease cPUR. Further studies are necessary to assess the long-term outcomes and improve understanding of postpartum voiding dysfunction.

## Introduction

Certain urogynecological functions such as urinary continence, fecal continence and bladder emptying are adversely affected immediately postpartum, yet these functions are rarely evaluated at this time^1,2^. Urinary retention is a common problem postpartum, with an estimated prevalence between 1.5 and 45%^3^, and can be either symptomatic or asymptomatic. Covert postpartum urinary retention (cPUR), first identified in 1961^4^, is diagnosed when a “post voiding residual volume >150 mL remains after spontaneous micturition of 150 mL”, and overt postpartum urinary retention (oPUR) is identified by the “inability to void spontaneously within 6 hours after vaginal delivery or within 6 hours after removal of an indwelling bladder catheter after cesarean section, requiring catheterization”^5^. The physiopathology of PUR remains unclear, although the role of progesterone on bladder compliance and the role of childbirth injury on bladder innervation are likely important factors^6,7^.

Contrary to cPUR, the postvoid residue (PVR) in healthy population is few studied. Choi *et al*. conducted a prospective study which reported 7,2% of voiding difficulty with obstruction in a population of 1415 non-pregnant women consulting in urology^8^. Median age was 62 years and median parity was 2. There is no epidemiologic study on prevalence of PVR in non-pregnant young women. In pregnant women, PVR rate is not known because of false positive diagnosis of PVR at ultrasound due to amniotic fluid^9^ and ethic impossibility to propose catheterization to healthy pregnant women.

A study from 2008 screening for cPUR in 154 consecutive parturients revealed a 36% rate of cPUR persistence at 72 h postpartum^10^, although a meta-analysis compiling 24 studies was unable to refine cPUR persistence due to the diversity of the included studies^3^. Long-term consequences of cPUR were not investigated in older studies and comparisons against control groups of patients with normal voiding functions were not carried out^11^. However, some research has shown that cPUR can have serious consequences. One study found 2 women with persistent PUR necessitating self-catheterization at home in a group of 114 cPUR patients^12^, whilst a retrospective study highlighted the occurrence of stress urinary incontinence and bladder overactivity in 50% of cases of persistent PUR^13^. Follow-up until 39 months of 55 women with persistent PUR confirmed rates of 10.4% of stress urinary incontinence, 8.3% of overactive bladder syndrome and 6.3% of subjective urinary symptoms^14^.

Screening and management for cPUR might prevent persistent PUR, long-term bladder catheterization and persistent voiding dysfunction. As noted by the authors of a meta-analysis, current data on cPUR is confused by the heterogeneity in the methodology of the included studies, including inclusion criteria, length and type of follow-up, and distinction between oPUR and cPUR, therefore further research has been encouraged, maintaining a homogeneity of definitions^15,16^.

In a recent prospective descriptive study conducted in our hospital, we found a high rate of cPUR of 52%, associated with risk factors identified in previous studies^16^: total labor length, duration at complete dilatation and instrumental delivery^17^. A high first voided volume was also associated with cPUR occurrence. To improve the management of women in postpartum, we proposed cPUR screening by ultrasound and adapted treatment. We established a systematic screening of cPUR to quickly restore voiding function and minimize the potential risk of urinary stress incontinence and bladder overactivity. In order to reduce the first void volume, and subsequent increased risk of cPUR, we established a systematic intermittent bladder catheterization 2 hours after delivery before patients go back to their rooms. The aim of this study was to evaluate the ability of systematic intermittent catheterization 2 hours after delivery to reduce the incidence of cPUR in a tertiary maternity center.

## Materials and Methods

This prospective monocentric “before-after” study was conducted in one university hospital center between May and September 2015. All parturients >18 years old admitted to the delivery room were eligible for this study. The women who subsequently underwent caesarean section were excluded. Women who had previous indwelling bladder catheterization during the third trimester of pregnancy or a history of surgery on the urinary tract were excluded. One research midwife was responsible for including patients into this study. All participants provided informed consent and received an information sheet. The institutional review board of the French college of obstetricians and gynecologists (CEROG OBS 2014-03-02) approved this study. All experiments were performed in accordance with relevant guidelines and regulations. This study is registered with ClinicalTrials.gov, number NCT02597413.

In the observational “no preventive catheterization” phase (group NPC), no systematic intermittent catheterization was performed. All women included in the preventive catheterization phase (group PC) underwent a systematic intermittent catheterization 2 hours after delivery. Theses two period were strictly separated in time in a sequential design. An intermittent catheterization was always performed before pushing in both groups. The primary endpoint was the rate of cPUR, defined as a post-void residual bladder volume >150 ml when voided volume is >150 mL. The post-void residual was assessed by an automatic ultrasound system BladderScan^®^ (Verathon, Schiltigheim, France). This method is noninvasive and easy to use. Previous studies have confirmed the reliability of automatic ultrasound system for detecting cPUR and its superiority compared to intermittent catheterization^18^. The first voided volume was recorded by midwives trained to measure post void residuals, immediately after spontaneous urination in the first 6 hours after delivery. For patients not voiding spontaneously within 6 hours of delivery, a BladderScan^®^ measure was performed to detect oPUR.

The secondary outcomes were sensation to void at 6 hours after delivery and the first spontaneous voided volume. Data concerning patient’s antecedents, labor, delivery and anesthesia were collected.

We assumed a reduction in the rate of cPUR of 60% with our new protocol (to decrease rate from 50% to 20%). We assigned two controls in group NPC for each patient in group PC. To achieve an alpha risk at 0.05% and a power of 80%, we needed to include 61 patients.

Data on baseline clinical characteristics are described in Table 1. All continuous variables are presented as mean+/− SD (standard deviation), all categorical data are presented as frequency, n (%,n). The rate of cPUR was compared with a two-sided Chi squared test. To assess the impact of confounding factors, a multiple logistic regression model was performed. The variables entered into this model were selected using a univariate logistic regression model (variable significant at the p < 0.2 level) and known risk factors. The continuous variables measured for the secondary endpoints were compared with Mann Whitney U test and categorical data with Chi squared test or Fisher’s exact test when conditions for Chi squared test were not valid. Analysis was performed according to the intention to treat principle. We performed prespecified subgroups analyses for mode of delivery. Tests of significance were two-sided with a 0.05 alpha risk. All data analysis was performed with R statistical software (Bell Laboratories, Lucent Technologies, http://www.r-project.org/).

**Table 1:** Baseline Patient Characteristics.

	**Group NPC (n** **=** **121)**	**Group PC (n** **=** **82)**	
Age (years) mean (SD)	30.2 (5.5)	29.8 (5.8)	p = 0.7
Previous cesarean section (%,n)	5% (6)	9% (7)	p = 0.31
Previous urinary incontinence (%,n)	6% (7)	0% (0)	p = 0.027
Previous recurrent urinary tract infection (%,n)	5% (6)	9% (7)	p = 0.12
Parity mean (SD)	2 (1)	2.1(1)	p = 0.63
**Complications during pregnancy**			
Preterm labor (%,n)	4% (5)	1% (1)	p = 0.23
Preeclampsia (%,n)	2% (3)	0% (0)	p = 0.15
Premature rupture of membranes (%,n)	0% (0)	0% (0)	
**Mode of delivery**			
Spontaneous vaginal delivery (%,n)	81% (98)	87% (71)	p = 0.3
Instrumental vaginal delivery (%,n)	19% (23)	13% (11)	
**Analgesia**			
No analgesia (%,n)	18% (22)	10% (8)	p = 0.31
Epidural analgesia (%,n)	80% (97)	89% (73)	—
Rachianesthesia	1% (1)	0% (0)	—
Rachianesthesia and epidural analgesia	1% (1)	1% (1)	—
**Labor management**			
Labor duration (min) mean (SD)	335 (181)	335 (183)	p = 0.84
Duration at complete dilatation (min) mean (SD)	45.2 (47)	79.3 (67)	p = 0.002
Duration of pushing (min) mean (SD)	13.5 (13)	12.3 (13)	p = 0.28
Number of intermittent catheterizations during labor mean (SD)	1.4 (0.8)	2.1 (1)	p < 0.001

## Results

Eighty-two patients were included in group PC between May and September 2015, with 121 women included in group NPC. This number was greater than expected due to rapidity to include patients and latency of study monitoring in closing inclusions during summer period. The baseline clinical characteristics are summarized in Table 1 and were similar between group NPC and PC. However, no patients in group PC had a history of urinary incontinence contrary to 7 in group NPC (p = 0.027). The average number of intermittent catheterizations during labor was 1.46 + /− 0.8 in group A and 2.12 + /− 1.07 in group PC (p < 0.01). Similar rates of instrumental delivery occurred between the groups. In group NPC, 23 of the 121 patients (19%) underwent instrumental delivery: 5 with vacuum, 16 with spatula and 2 with forceps; while 11/82 (13%) patients in group PC had an instrumental delivery: 1 with vacuum, 9 with spatula and 1 with forceps.

The analysis of the global population of 203 women found duration of pushing as a potential risk factor of cPUR (p = 0.04). Total duration of labor and instrumental delivery were not risk factors in global population (p = 0.159 and p = 0.1 respectively). Increasing number of catheterizations during labor was potentially a protective factor, but was not statistically significant (p = 0.26).

**Figure 1:**
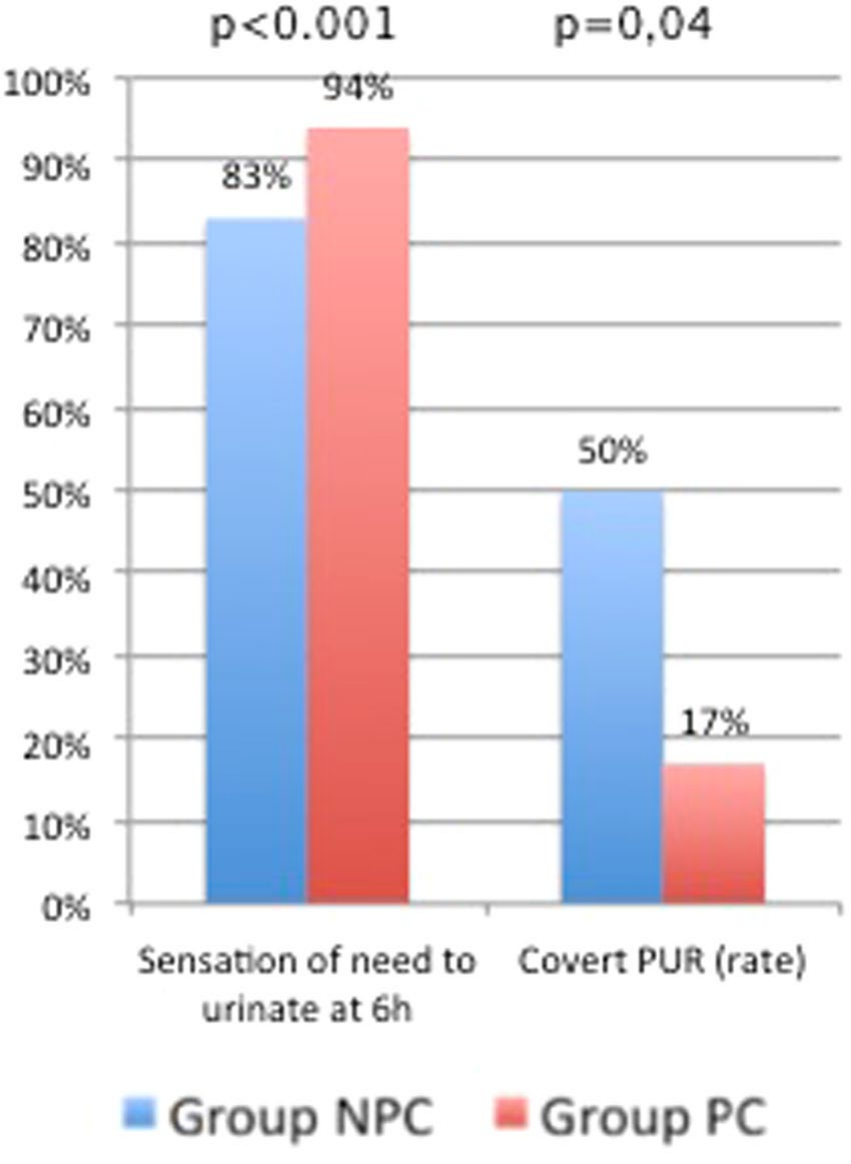
Rates of sensation of need to urinate at 6 h and cPUR in groups NPC and PC in overall population.

**Figure 2:**
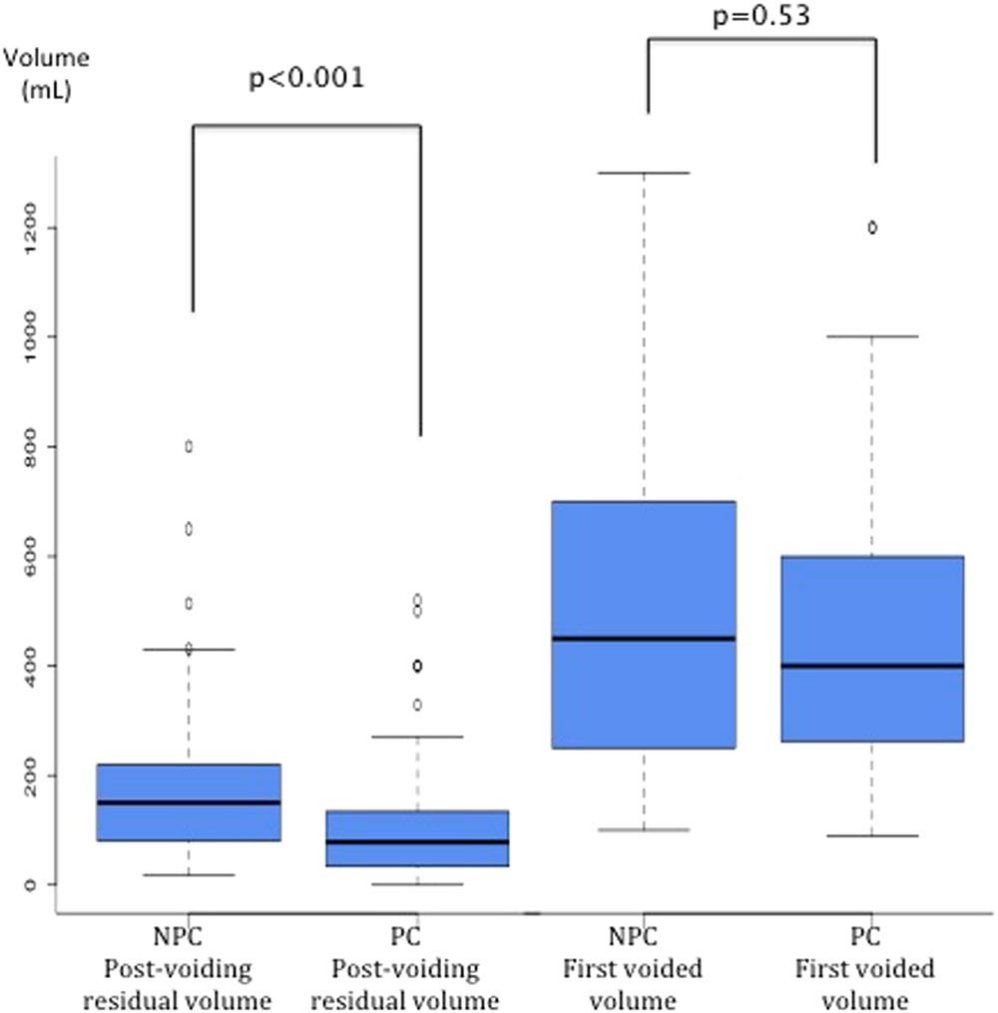
Results for Post-voiding residual volume and first voided volume in groups NPC and PC in overall population.

The rate of cPUR was higher in group NPC (50%, 60/121) than in group PC (17%, 14/82) (OR = 0.21, 95% Confidence Interval (CI) [0.11;0.41], p < 0.001*) (Fig. 1). The difference in rate of cPUR between groups was also significant in multivariate analysis considering labor duration, pushing duration, voiding sensation at 6 hours and the number of intermittent catheterizations during labor. The adjusted OR for cPUR occurrence was 0.27 (95% CI [0.13;0.58]; p < 0.001). The mean post-voiding residual was 38% lower in group PC than in group NPC (105 + /− 111 mL in group PC versus 171 + /− 127 mL in group NPC p < 0.001) (Fig. 2).

The first spontaneous voided volume was similar in the two groups (481 + /− 277 mL in group NPC vs 457 + /− 250 mL in group PC p = 0.53) (Fig. 2). More patients in group PC felt voiding desire than in group NPC (83%, 101/121 in group NPC vs 94%, 77/82 in group PC, p = 0.03*)(Fig. 1). This difference is also significant in multivariate analysis (OR = 0.38, 95% CI [0.15;0.97], p = 0.04*).

**Figure 3:**
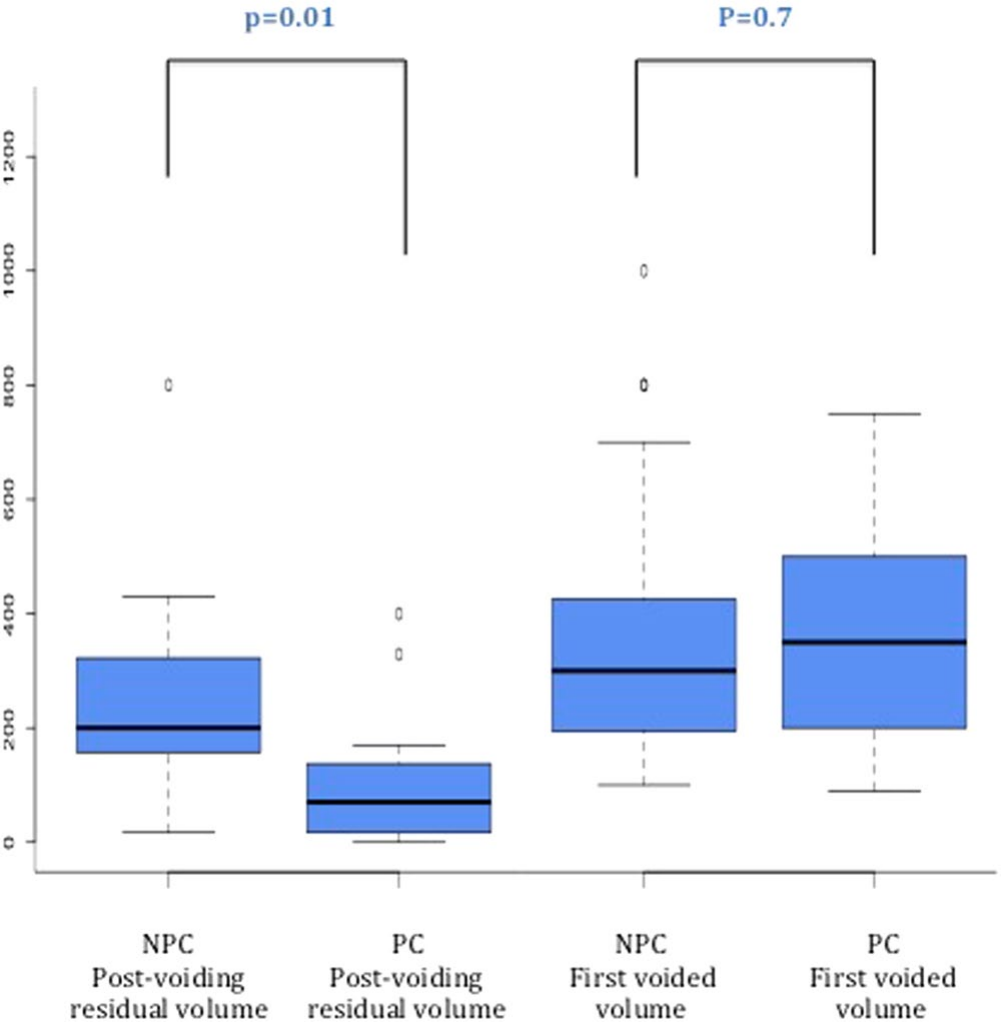
Results for Post-voiding residual volume and first voided volume in groups NPC and PC in instrumental-delivery population.

The decrease in the rate of cPUR remained significant in subgroup analysis for mode of delivery. In the subgroup of patients who underwent instrumental delivery, the rate of cPUR was lower in group PC (18%, 2/11) than in group NPC (65%, 15/23) (p = 0.02). The mean volume of post void residual in this subgroup was 240 + /−164 mL in group NPC and 115 + /− 134 mL in group PC (p = 0.01*)(Fig. 3). In this subgroup, 74% (17/23) of NPC patients felt the desire to void at 6 hours after delivery compared to 82% (9/11) in group PC (p = 0,61). In the subgroup of patients with spontaneous vaginal delivery, the rate of cPUR was also significantly lower in group PC: 46% (45/98) in group NPC vs 17% (12/71) in group PC p < 0.01).

## Discussion

### Main Findings

Systematic intermittent catheterization 2 hours post-delivery was effective at decreasing cPUR by 47% in women undergoing instrumental deliveries and 29% for women having spontaneous vaginal delivery. It is notable that preventive intermittent catheterization decreased cPUR to the same level in the instrument deliveries subgroup, despite this subgroup being much more at risk for developing cPUR. Whilst patients undergoing instrumental deliveries in group NPC had increased cPUR in comparison with those having spontaneous vaginal delivery, there was no difference between these subgroups in the PC group (17 vs 18%).

The rate of cPUR in our study before establishment of systematic catheterization was 50%, the higher end of the scale for cPUR found in the literature^17^. The rate of cPUR and the first voiding volume were similar to those reported in a recent prospective study^16^. There is no published data on the cPUR rate in asymptomatic young women, during our outside pregnancy. In the literature, risk factors associated with PUR are labor duration, parity, mode of delivery, perineal tears and epidural analgesia^5,12,15,19,20^. In our first study, we found similar prognostic factors, with duration at complete dilatation, total labor duration and instrumental delivery associated with cPUR occurrence^15^. However, in this study, epidural analgesia and parity was not associated with cPUR. This may be explained by the high rate of epidural analgesia in our center (80% in group NPC and 89% in group PC).

### Strengths and Limitations

In this study we defined cPUR according to the recently proposed definition by Yip *et al*.^5^, meaning that our study can be compared against future research using these same parameters. Nevertheless, this study has some limitations. The main weakness was the comparability of the different groups due to the study design, including the number of catheterizations during labor. The duration at complete dilatation is also greater in group PC; however, as the effect of this difference would be in favor of the null hypothesis, it does not bias the interpretation of our results.

### Interpretation

This study was conceived following the findings of high rate of cPUR^17^ in our center in order to improve early rehabilitation in postpartum as recommended by WHO^21^. Our hypothesis was that bladder overdistension in postpartum associated with bladder atony due to instrumental delivery or epidural analgesia induce high rates of cPUR. The aim was to improve voiding sensation in postpartum to decrease the risk of cPUR. Even following systematic intermittent catheterization, the first voided volume remains high in our study, with an average first voided volume of 457 + /−250 mL, but an improvement in sensation to void 6 hours after delivery was observed. The decrease of cPUR is probably multifactorial.

Firstly, the systematic catheterization did not result in a decrease in the first voided volume but it could lessen the duration of high and harmful bladder volume. In consequence, detrusor lesions may not have appeared in the first 6 hours. Secondly, whilst the management of labor and analgesia in the delivery room was unchanged between the two periods, we nevertheless observed an increase of the number of intermittent catheterizations during labor. This increase is probably the result of awareness by midwifes and health staff of our study. This more regular emptying of the bladder may have reduced bladder traumatism secondary to labor and compression by fetus head and could participate in the decrease of cPUR rate. However, multivariate logistic regression confirmed the beneficial role of the postpartum preventative catheterization. Thirdly, 17% of women still suffered from cPUR. In the global population, pushing duration is a risk factor of cPUR. A longer expulsion with perineal distension can cause more pelvic neuropathy, explaining persistence of cPUR and the failure of the catheterization procedure as a preventing measure.

We cannot assess the safety of systematic intermittent catheterization 2 hours after delivery concerning bacteriuria. In our previous observational study, the rate of bacteriuria was 13%^17^. Only Body Mass Index was associated with bacteriuria, whilst cPUR occurrence and number of catheterizations were not associated with bacteriuria in postpartum. Therefore we did not study bacteriuria in the PC phase. The majority of other studies that investigate the bacteriuria in postpartum have also found number of catheterizations is not a risk factor, although some studies show the reverse^22–25^.

Our study demonstrates that cPUR is preventable in postpartum for the majority of patients, suggesting there is a problem in bladder management in the postpartum period. Efficacy of the catheterization protocol was confirmed even in high-risk patients with instrumental delivery. Reclassifying cPUR as a physiological rather than pathological event could be reconsidered once more follow-up data is available on cPUR consequences to identify women at risk of persistent PUR. These patients could be reconvened in the future for clinical evaluation of urinary functions.

## Conclusion

In summary, we have shown that the establishment of a systematic intermittent catheterization could decrease the rate of cPUR. These results might improve early rehabilitation and understanding of postpartum bladder dysfunction. Available long-term data suggests low impact of cPUR on urogynecological functions but need to be better explored before modification of daily practice.

Homogeneity of the definition of cPUR is still a problem and some authors have suggested changing the definition of Yip *et al*.^20^. In their meta-analysis, Mulder *et al*. caution that the current knowledge was insufficient to conclude that cPUR is harmless and that it is merely a physiological condition. We agree with Mulder *et al*. that cPUR could be reconsidered as a physiological condition when new data on clinical consequences become available, and we encourage use of the cPUR definition of Yip *et al*., used here, in further studies, particularly with a longer follow-up.

### Data Availability

The datasets generated during and analysed during the current study are available from the corresponding author on reasonable request.

### Details of ethics approvals

The Ethic committee of the French College of Obstetricians and Gynecologists approved this study: CEROG OBS 2014-03-02.
